# Direct Gaze Partially Overcomes Hemispatial Neglect and Captures Spatial Attention

**DOI:** 10.3389/fpsyg.2018.02702

**Published:** 2019-01-15

**Authors:** Miguel Leal Rato, Inês Mares, Diana Aguiar de Sousa, Atsushi Senju, Isabel Pavão Martins

**Affiliations:** ^1^Hospital Prof. Doutor Fernando Fonseca, Amadora, Portugal; ^2^Centre for Brain and Cognitive Development, Department of Psychological Sciences, Birkbeck, University of London, London, United Kingdom; ^3^Department of Neurosciences and Mental Health, Neurology, Hospital de Santa Maria, University of Lisbon, Lisbon, Portugal; ^4^Language Research Laboratory, Instituto de Medicina Molecular, University of Lisbon, Lisbon, Portugal

**Keywords:** hemispatial neglect, direct gaze, averted gaze, visuospatial attention, unconscious perception

## Abstract

Direct gaze has been shown to be a particularly important social cue, being preferentially processed even when unconsciously perceived. Results from several visual search tasks further suggest that direct gaze modulates attention, showing a faster orientation to faces perceived as looking toward us. The present study aimed to analyze putative modulation of spatial attention by eye gaze direction in patients with unilateral neglect. Eight right hemisphere stroke patients with neglect performed a target cancelation paradigm. Patients were instructed to cross all open-eyed pictures amidst closed eyed distractors. Target images were either in direct or averted gaze. Participants performed significantly better when observing targets with direct gaze supporting the hypothesis that this gaze direction captures attention. These findings further suggest that perception of direct gaze is able to diminish the visuospatial impairment seen in neglect patients.

## Introduction

Detecting salient stimuli in the environment is crucial for survival and adaptation, allowing an individual to orient to possible sources of threat or to relevant objects. Saliency refers to a stimulus’ distinctive sensorial properties or behavioral importance in relation to others. In humans, direct gaze could be a social cue of particular saliency due to its significance in communication and non-verbal social interaction (von Grünau and Anston, 1995).

Direct gaze has been shown to modulate several concurrent cognitive processes such as face encoding and retrieval (Conty and Grèzes, 2012), emotion processing (for a review see Rigato and Farroni, 2013), gender discrimination (Macrae et al., 2002; Vuilleumier et al., 2005), retrieval of semantic information (Macrae et al., 2002) and pro-social behavior (Bateson et al., 2006). Furthermore, direct gaze is preferentially detected in visual search tasks when compared with other gaze directions (von Grünau and Anston, 1995; Senju et al., 2005), a preference that occurs even in express saccades as observed in reflexive orienting (Mares et al., 2016). This preference for direct eye gaze is present from birth as observed in newborn babies (Farroni et al., 2002). Privileged perception of direct gaze occurs even under unconscious stimulus presentation, as shown in a paradigm using a form of binocular rivalry, continuous flash suppression (CFS; Stein et al., 2011). In this paradigm, direct gaze was shown to break suppression faster than averted gaze, with a concomitant modulation of neural responses suggesting that faces with direct gaze are processed effectively under an unconscious condition (Yokoyama et al., 2013). Furthermore, enhanced amygdala activation for direct gaze was found in a patient with complete cortical blindness (Burra et al., 2013). This line of research suggests that direct gaze can be partially processed even when not consciously perceived. In this case, some residual processing of direct gaze could still modulate orienting even in patients with damaged attention mechanisms, as occurs in hemispatial neglect (HSN).

HSN commonly occurs following right hemisphere lesions and is characterized by contralesional spatial defects (such as deficits in saliency coding, spatial attention and visuospatial short-term memory), alongside with non-spatial defects (reorienting, target detection, and arousal/vigilance deficits; Vallar, 1998). The classic, viewer-centered (egocentric), neglect (Medina et al., 2009) occurs mainly after asymmetric or focal brain damage, most frequently caused by stroke in the right hemisphere (Hillis, 2013), particularly in the inferior parietal lobule, superior temporal gyrus and/or inferior frontal gyrus, leading to deficits on the left side of space (Corbetta and Shulman, 2011; Corbetta, 2014). A study by Azouvi et al. (2002) has shown that about 85% of subacute right hemisphere stroke patients presented some degree of unilateral neglect, which was considered as clinically significant (moderate to severe) in 36.2% of cases. The presence of HSN was task dependent, as tasks including a strong visual component were the most sensitive to the spatial defect of HSN, and the automatic rightward orientation bias “(i.e., the spontaneous tendency to orient toward the right hemifield)” seemed to be the best indicator of unilateral neglect.

The neural mechanisms underlying HSN spatial deficit can be dynamically modulated by either endogenous or exogenous signals, creating a complex interaction between attention, movement and arousal (Corbetta, 2014; Duclos et al., 2014). Interestingly, stimuli do not need to be consciously perceived by the HSN patient, who can use the information provided by peripheral cues to orient their attention toward the neglected space, even without conscious awareness of the presented stimuli (Wansard et al., 2015). This has been often observed for instance with emotional stimuli (see Domínguez-Borràs et al., 2012 for a review). Gaze in particular has been shown to be able to modulate attention in neglect patients when presented centrally (Maravita et al., 2007), decreasing peripheral target detection on the contralateral hemispace.

Despite this, no study to our knowledge has shown a modulatory effect of direct gaze on attention throughout the visual space in patients with HSN. The presence of such an effect would be supported by behavioral studies that have shown that perception of direct gaze still occurs when attention is diminished in typical participants (Yokoyama et al., 2014). Thus, we hypothesize that direct gaze can be processed even when individuals are unable to direct their attention toward it. To test this hypothesis, we investigated direct gaze detection in patients with HSN. We predict that direct gaze processing can take place even when the HSN patients are unable to attend to it, which should result in a better performance in detecting direct gaze than averted gaze.

## Materials and Methods

### Patients

Nineteen patients (7 females; age, range: 41–84, *M* = 64.32 ± 12.96 years old; education, range: 4–13, *M* = 6.68 ± 3.49 years) with right hemisphere acute stroke admitted to a Stroke Unit were included for the initial assessment. Patients were excluded if they presented with significantly altered mental state as assessed by clinical evaluation, any major medical comorbidity, a significant speech or comprehension impairment or if they were in a state of non-cooperation. All patients signed an informed consent previously to participation and this study was reviewed and given authorization to start by the local Ethics Committee (Centro Hospitalar Lisboa Norte – Faculdade de Medicina de Lisboa ).

### HSN Assessment

All patients were assessed with a bedside HSN test battery including tasks of star cancelation, line crossing, figure copying, and menu reading adapted from the Behavioral Inattention Test (BIT, Wilson et al., 1987), presented sequentially. HSN was defined as any left skewed visuospatial defect identified in either the star cancelation or line crossing tasks.

### Experimental Procedure

Patients with HSN performed a cancelation task consisting of an array with images of open and closed eyes. Patients were asked to search and mark by crossing over the stimulus all open-eyed stimuli (either in direct or averted gaze) amongst closed-eyed distractors. Fourteen targets and distractors were distributed across a standard white horizontal 21 × 29.7 cm (A4) sheet of paper, centered relative to each patient’s body midline, in an unstructured pseudo-random array, in order to increase the sensitivity of the tasks (Azouvi et al., 2002). All faces were laterally oriented to the right or left, to avoid low level visual confounds such as face symmetry (George et al., 2001). Each participant performed four randomized trials corresponding to four different conditions varying target gaze direction (direct and averted gaze) and overall face orientation (right and left).

The sheets were randomized by an outside person by printing in random order and manually shuffling the sheets before participant inclusion. Allocation was secured by keeping each set of sheets in an opaque envelope until bedside examination.

**FIGURE 1:**
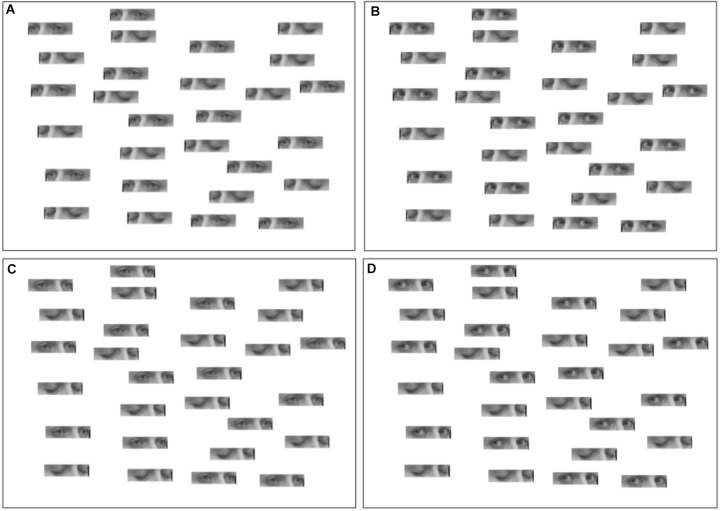
Tasks. **(A)** Faces oriented to the left, eyes in averted gaze. **(B)** Faces oriented to the left, eyes in direct gaze. **(C)** Faces oriented to the right, eyes in averted gaze. **(D)** Faces oriented to the right, eyes in direct gaze.

Meaning, a patient would be shown a random sequence of 4 trials, one with direct gaze and right face orientation, another with direct gaze and left face orientation and the corresponding for averted gaze (Figure 1). Participants had no time limit to complete each task.

Patients were free to move their heads in relation to the presented sheets. Although this could possibly be a confounding factor (as the side of the sheet that is in the left hemispace will no longer necessarily be in the left hemifield), we know that patients with neglect tend to avoid exploring the left hemispace, due to an ipsilesional gaze bias (Fruhmann Berger et al., 2008). Thus we expect an overall neglect of the left hemispace irrespective of hemifield.

Trials with oriented faces to the right and left were merged for each gaze condition. Accuracy was assessed as the total number of open-eyed stimuli identified, with a larger number indicating a smaller spatial defect.

### Statistical Analysis

To analyze the effect of gaze direction across the visual field, targets were divided into four vertical areas of equal size. Two way repeated measures analysis of variance (ANOVA) were performed, with gaze direction (direct and averted) and position (four positions from the left to the right) as factors for hits, false alarms and d’. d’ is a measure of accuracy, with hits defined as correctly crossed open eyes, and false alarms defined as incorrectly crossed closed eyes. Hit and false alarm rates of one were corrected by calculating 1-1/(2^∗^number of possible hits/false alarms) and zero by calculating 1/(2^∗^ number of possible hits/false alarms). Additional *post hoc* analyses (two-tailed *t*-tests) were performed as required. A possible effect of gaze cueing was also analyzed for targets with averted gaze using a two-tailed *t*-test. Effect sizes for dependent *t*-tests were calculated using the formula proposed in Eq. (3) of Dunlap et al. (1996).

## Results

Eight of the 19 patients assessed were found to have HSN (3 females; age, range: 53–80, *M* = 65.75 ± 8.61 years old; education, range: 4–12, *M* = 5.37 ± 2.77 years). There was no significant difference between non-HSN and HSN patients regarding age, gender distribution, years of education, or time from stroke until assessment.

**FIGURE 2:**
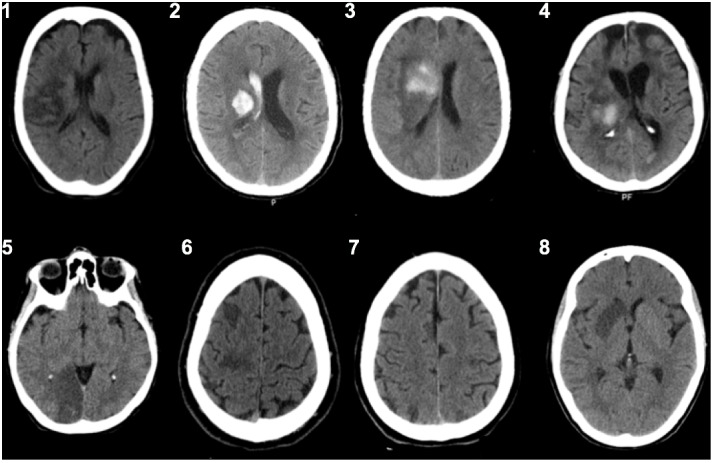
CT scans from each patient (patient ID 1–8, see Table 1) at time of assessment.

Demographic characteristics and clinical data of the studied HSN population are shown in Table 1 and CT scan results in Figure 2.

**Table 1:** Demographic characteristics and clinical data of the studied neglect population and main results (I = Ischaemic, H = Hemorrhagic, AG = Averted Gaze, DG = Direct Gaze).

Patient ID	Education (years)	Days since stroke	Stroke^∗^	Hit rate		False alarm rate		d’	
				AG	DG	AG	DG	AG	DG
1	4	2	I	0.32	0.57	0.11	0.02	0.78	2.28
2	4	4	H	0.61	0.71	0.07	0.11	1.74	1.81
3	4	4	H	0.21	0.25	0.18	0.07	0.13	0.79
4	4	5	H	0.21	0.21	0.02	0.04	1.31	1.01
5	12	5	I	0.93	0.98	0.04	0.02	3.27	4.20
6	6	5	I	0.79	0.79	0.02	0.02	2.89	2.89
7	4	4	I	0.29	0.39	0.32	0.43	-0.10	-0.09
8	5	2	I	0.11	0.21	0.14	0.07	-0.17	0.67


A positive correlation was found between the scores on the line cancelation task, used to assess HSN, and the number of targets identified in direct and averted gaze (*r* = 0.84, *n* = 8, *p* = 0.009 and *r* = 0.88, *n* = 8, *p* = 0.004, respectively). Furthermore, the difference between accuracy in direct and averted gaze correlated negatively with the time from stroke until assessment (*r* = -0.79, *n* = 8, *p* = 0.019).

**FIGURE 3:**
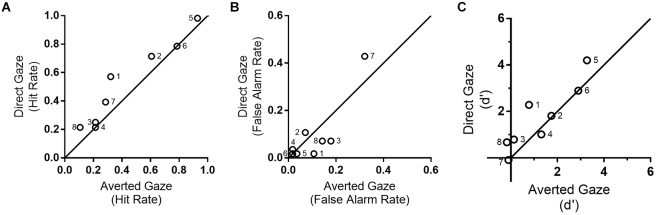
**(A)** Hit rate in direct (vertical axis) and averted gaze (horizontal axis). Six out of eight patients detected more targets in direct than averted gaze, with the remaining two showing no difference between conditions; **(B)** False alarm rate for direct (vertical axis) and averted gaze tasks (horizontal axis); **(C)** d’ analysis. See Supplementary Figure 1 for hit rate separated by head orientation of the stimuli. The numbers on each point of the graphs correspond to patient ID (see Table 1).

Hit rates were higher for direct gaze (*M* = 0.52 ± 0.24) than for averted gaze [*M* = 0.45 ± 0.25; *F*(3,21) = 8.61, *p* = 0.022, $n_{p}^{2}$ = 0.55]. A main effect of position was also significant [*F*(3,21) = 11.44, *p* < 0.001, $n_{p}^{2}$ = 0.62], with decreasing hit rates toward the left hemispace (*p* < 0.043), with exception of the two most leftward areas which did not differ (*p* = 0.028; from right to left, 1st, *M* = 0.82 ± 0.16; 2nd, *M* = 0.50 ± 0.37; 3rd, *M* = 0.38 ± 0.38; 4th, *M* = 0.23 ± 0.24). As before no interaction between stimuli position and gaze direction was found [*F*(3,21) = 1.45, *p* = 0.26, $n_{p}^{2}$ = 0.17]. Figures 3A,B show the hit rate and the false alarm rate for averted and direct gaze, respectively. A version of this graph (Supplementary Figure 1) showing the hit rate for averted and direct gaze divided by left or right head orientation of the stimuli is available as Supplementary Material.

Regarding false-alarms, there was an interaction between gaze direction and stimuli position [*F*(3,21) = 5.33, *p* = 0.007, $n_{p}^{2}$ = 0.43]. There was a trend for more false alarms for averted gaze trials (*M* = 0.30 ± 0.23) compared with direct gaze (*M* = 0.16 ± 0.12) in the most rightward position [*t*(7) = 2.32, *p* = 0.05, Cohen’s d = 0.59], with no difference for the remaining positions (*p* > 0.17). Note that false alarms were not higher for direct than for averted gaze, which shows that the higher hit rates for direct gaze condition is not based on overall response bias (i.e., to cross out any stimuli).

For accuracy, as assessed with d’, we observed a significant main effect of position [*F*(3,21) = 6.05, *p* = 0.004, $n_{p}^{2}$ = 0.464], with a difference between the most extreme right area (*M* = 1.92 ± 1.05), and marginally the second (*p* = 0.07, *M* = 1.18 ± 1.45), and significantly the remaining most leftward areas (*p* < 0.04; *M* = 1.00 ± 1.38, *M* = 0.371 ± 0.81, respectively) (see Figure 3C ). We also observed a trend for larger accuracy for direct gaze (*M* = 1.26 ± 1.04), compared to averted gaze (*M* = 0.98 ± 1.03), which did not reach significance [*F*(1,7) = 4.39, *p* = 0.074, $n_{p}^{2}$ = 0.39]. Interaction between position and gaze direction was not significant either [*F*(3,21) = 0.55, *p* = 0.65, $n_{p}^{2}$ = 0.07].

No effect of gaze cueing toward the neglected hemispace was observed for d’ [*t*(7) = 0.77, *p* = 0.47, Cohen’s d = 0.20], with no differences in accuracy found between faces in averted gaze toward the left (*M* = 1.29 ± 1.18%) or the right (*M* = 1.02 ± 1.44%). Similarly, no difference were observed when using hit rates [*t*(7) = -0.18, *p* = 0.86, Cohen’s d = -0.03]. Main results are summarized in Table 1.

## Discussion

The current study tested the hypothesis that residual processing of direct gaze is sufficient to lead to an increased detection of targets that would otherwise be unattended. The current findings support this hypothesis as participants detected a higher proportion of faces with direct gaze than faces with averted gaze. This result reflects differences in attentional capture.

This replicates previous studies that have shown that residual abilities in sensorial processing can modulate attention toward the contralesional stimuli if the perceived stimuli are salient. Socially or biologically relevant stimuli such as faces have been shown to be partially analyzed without attention (Vuilleumier, 2000). Using fMRI and ERP methodologies, unconscious face perception in HSN patients has further been shown to elicit V1 activations and N170 face specific components suggesting that visual processing still occurs in the absence of awareness (Vuilleumier et al., 2001). Furthermore, emotional expressions have been found to modulate spatial attention and orienting in HSN patients (for a review see Domínguez-Borràs et al., 2012). Direct gaze in particular has been shown to be partially processed in the absence of conscious perception, both in a patient with blindsight (Burra et al., 2013) and in paradigms (e.g., CFS) that use binocular rivalry to present stimuli unconsciously (Stein et al., 2011; Yokoyama et al., 2013).

Nonetheless, previous studies failed to demonstrate an advantageous effect of direct gaze in HSN. Vuilleumier (2002) tested the effect of gaze direction on attention in HSN patients by using an extinction paradigm, where patients’ ability to detect contralesional stimuli is diminished by a simultaneous display on the ipsilesional field. Vuilleumier’s (2002) study did not find an attention capture effect of direct gaze when displayed on the left visual field. On the other hand, a congruent gaze direction, meaning, gaze directed toward the left, was shown to cue attention to the contralesional spatial hemifield, leading the authors to conclude that gaze information is not extracted unconsciously or “preattentively” in HSN. Results from the present study seem to oppose both these findings with a clear attention capture of direct gaze occurring throughout the visual space, and in particular in the left hemispace, in the absence of a possible cueing effect of faces with averted gaze toward the left. The use of an extinction paradigm in Vuilleumier’s (2002) study could account for the different results. It is possible that while a gaze cueing effect in HSN might be specific to the extinction symptoms, a cancelation task might be more sensitive to evaluate the effect of direct gaze in the broader neglect deficit.

Our study is the first to demonstrate that direct gaze enhances target detection in the HSN patients, diminishing the visuospatial impairment associated with HSN. These unconscious effects of direct gaze have been proposed to result from a ‘fast’ pathway, mediated by subcortical structures including the superior colliculus, pulvinar, and amygdala (Senju and Johnson, 2009).

Individuals with lesions involving the medial cortico-subcortical networks may suffer from more severe HSN symptoms and are more likely to develop chronic spatial neglect. Chen et al. (2012) found that this might explain the degree of efficacy of HSN treatments, such as prism adaptation treatment. These medial temporal regions might even provide critical support for neural or chemical plasticity in spontaneous recovery. Moreover, due to the importance of the pulvinar to certain core mechanisms of attention, for instance the unconscious processing of salient stimuli such as faces (Troiani and Schultz, 2013), it may play an important role in this syndrome and its integrity might be related to a better prognosis and be predictive of treatment success.

The presence of a “preattentive” effect of eye contact on patients with HSN might be mediated by this pathway, in which case a lesion in one of its core structures might abolish such an effect. Future studies analyzing the effect of direct gaze in HSN patients with neglect due to pulvinar damage could clarify the role of this pathway in gaze processing.

As for limitations, we did not include a control group, as we expected a task ceiling effect in participants without neglect. Nevertheless, we recognize that this is a potential flaw in design. It would need to be addressed in a future study, ideally including a control group without neglect, but with whom a cancelation task will measure a non-ceiling and meaningful effect. Additionally, as patients did not have a time limit to complete our tasks, latency or speed of stimuli completion was not evaluated. Taking into account the small sample size, future studies are needed to replicate these preliminary findings in a larger sample of patients with neglect.

## Conclusion

The current study suggests that direct gaze is processed pre-attentively, helping to partially overcome spatial deficits in HSN. Furthermore, it provides converging evidence of the saliency of direct gaze when compared with other gaze directions by using a common organic lesion paradigm. The benefit of direct gaze (putatively due to its inherent saliency) was not specific for the left hemifield. Further studies will be beneficial to understand the underlying neural structures of the effect of direct gaze on visuospatial attention or how it can improve the management of chronic HSN patients.

## Ethics Statement

The project and its protocol were approved by the Ethics Committee of the Hospital de Santa Maria, Centro Hospitalar Lisboa Norte. All subjects gave written informed consent in accordance with the Declaration of Helsinki.

## Author Contributions

MLR and IM revised the literature. MLR, IM, and IPM conceived and designed the experiments. MLR and DAdS collected the data. MLR, IM, and AS performed the analysis. MLR and IM wrote a first draft of the manuscript that was revised by DAdS, AS, and IPM. All authors read and approved the final manuscript.

## Conflict of Interest Statement

The authors declare that the research was conducted in the absence of any commercial or financial relationships that could be construed as a potential conflict of interest. The reviewer GV and handling Editor declared their shared affiliation at the time of review.
