# Supplementary figures and images for: Direct Gaze Partially Overcomes Hemispatial Neglect and Captures Spatial Attention
Source: Front Psychol. 2019 Jan 15;9:2702. doi: 10.3389/fpsyg.2018.02702 (PMC6340963; doi:10.3389/fpsyg.2018.02702)

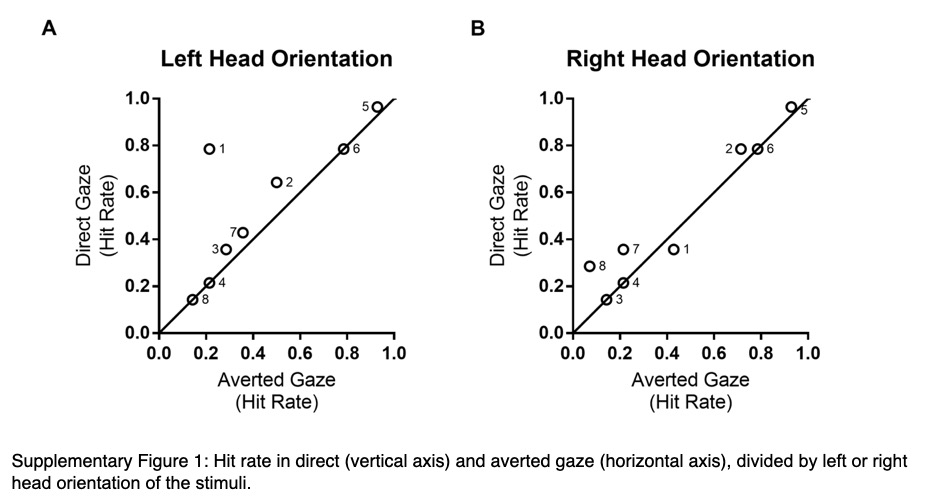

Supplement: Supplementary file 1 [file Image_1.jpg]
